# Palmitoylation regulates the intracellular trafficking and stability of c-Met

**DOI:** 10.18632/oncotarget.8706

**Published:** 2016-04-12

**Authors:** David T. Coleman, Alana L. Gray, Steven J. Kridel, James A. Cardelli

**Affiliations:** ^1^ Department of Microbiology and Immunology and Feist-Weiller Cancer Center, Louisiana State University Health Sciences Center-Shreveport, LA 71130, USA; ^2^ Department of Cancer Biology, Wake Forest University School of Medicine, Winston-Salem, NC 25157, USA

**Keywords:** c-Met, palmitoylation, intracellular trafficking, cancer, click chemistry

## Abstract

c-Met is a receptor tyrosine kinase whose activity can promote both mitogenic and motogenic phenotypes involved in tissue development and cancer progression. Herein, we report the first evidence that c-Met is palmitoylated and that palmitoylation facilitates its trafficking and stability. Inhibition of palmitoylation reduced the expression of c-Met in multiple cancer cell lines post-transcriptionally. Using surface biotinylation, confocal microscopy, and metabolic labeling we determined that inhibition of palmitoylation reduces the stability of newly synthesized c-Met and causes accumulation at the Golgi. Acyl-biotin exchange and click chemistry-based palmitate labeling indicated the c-Met β-chain is palmitoylated, and site-directed mutagenesis revealed two likely cysteine palmitoylation sites. Moreover, by monitoring palmitoylation kinetics during the biosynthesis and trafficking of c-Met, we revealed that stable palmitoylation occurs in the endoplasmic reticulum prior to cleavage of the 170 kDa c-Met precursor to the mature 140 kDa form. Our data suggest palmitoylation is required for egress from the Golgi for transport to the plasma membrane. These findings introduce palmitoylation as a critical modification of c-Met, providing a novel therapeutic target for c-Met-driven cancers.

## INTRODUCTION

The c-Met receptor tyrosine kinase (RTK) is involved in embryonic development, tissue remodeling, and, when dysregulated, cancer progression [1]. The c-Met protein is synthesized initially as a 170 kDa single chain precursor that is co-translationally glycosylated and cleaved in the Golgi into a disulfide-linked α-chain (50 kDa) and β-chain (140 kDa) [2]. The receptor is trafficked through the Golgi to the plasma membrane where its levels are maintained at steady-state internalization and degradation rates, the kinetics of which are increased upon activation with ligand [3–6]. In addition to internalization, the extracellular domain of c-Met can be cleaved by a disintegrin and metalloproteinase (ADAM) family proteases and shed [7–9]. Binding of hepatocyte growth factor (HGF) initiates autophosphorylation and downstream signaling cascades promoting growth, survival, and motility that is essential for development and tissue repair. When unrestricted due to gene amplification, activating mutations, or heightened ligand autocrine and paracrine secretion, the signaling pathway becomes oncogenic [10–14].

c-Met is frequently overexpressed or mutated in cancers and this coincides with disease progression [14]. Receptor overexpression alone can cause homodimerization and constitutive activation independent of ligand binding [15]. Aberrant activation of the c-Met signaling pathway can cause unrestricted proliferation and survival, as well as heightened invasive potential [16–19]. Moreover, recent findings have demonstrated a frequent role for c-Met as a mediator of resistance to targeted therapies [20–24]. Reducing the expression and inhibiting the activity of c-Met is a clear target for preventing progression or therapeutic resistance of a number of cancers [25]. Understanding the regulation of c-Met levels will aid in the development of novel therapeutics.

Our laboratory and others have found a connection between fatty acid synthase (FASN) activity and c-Met [26]. Inhibition of FASN activity lowers total c-Met protein levels, and this reduction can be prevented by exogenous palmitate indicating the fatty acid is necessary for stability of c-Met [27]. Several groups have demonstrated that inhibition of FASN activity can prevent protein palmitoylation. Moreover, FASN expression can promote the palmitoylation of proteins and therefore influence their activity *in vivo* [28–30]. Palmitate can be attached to proteins by palmitoyl acyltransferases (PATs) via an N-terminal amide bond or cysteine-linked thioester bonds (S-palmitoylation). Palmitoylation and other lipid modifications, such as myristoylation (14-carbon amide-linked myristate) and prenylation (cysteine-linked farnesyl or geranylgeranyl groups), are candidate drug targets [31, 32]. A number of cancer-related signaling proteins require palmitoylation for their spatial regulation; but little has been shown for single-transmembrane spanning receptor tyrosine kinases [32–36]. The lack of a strong consensus sequence for S-palmitoylation and the intractable nature of traditional radiolabeling experiments have hampered the study of protein acylation [37, 38]. Given our observations, we hypothesized that palmitoylation stabilizes c-Met. Herein, we employ innovative techniques for acylated protein detection to test our hypothesis and determine the role of palmitoylation in c-Met expression and stability.

## RESULTS

### Inhibition of palmitoylation lowers total c-Met protein levels

**Figure 1:**
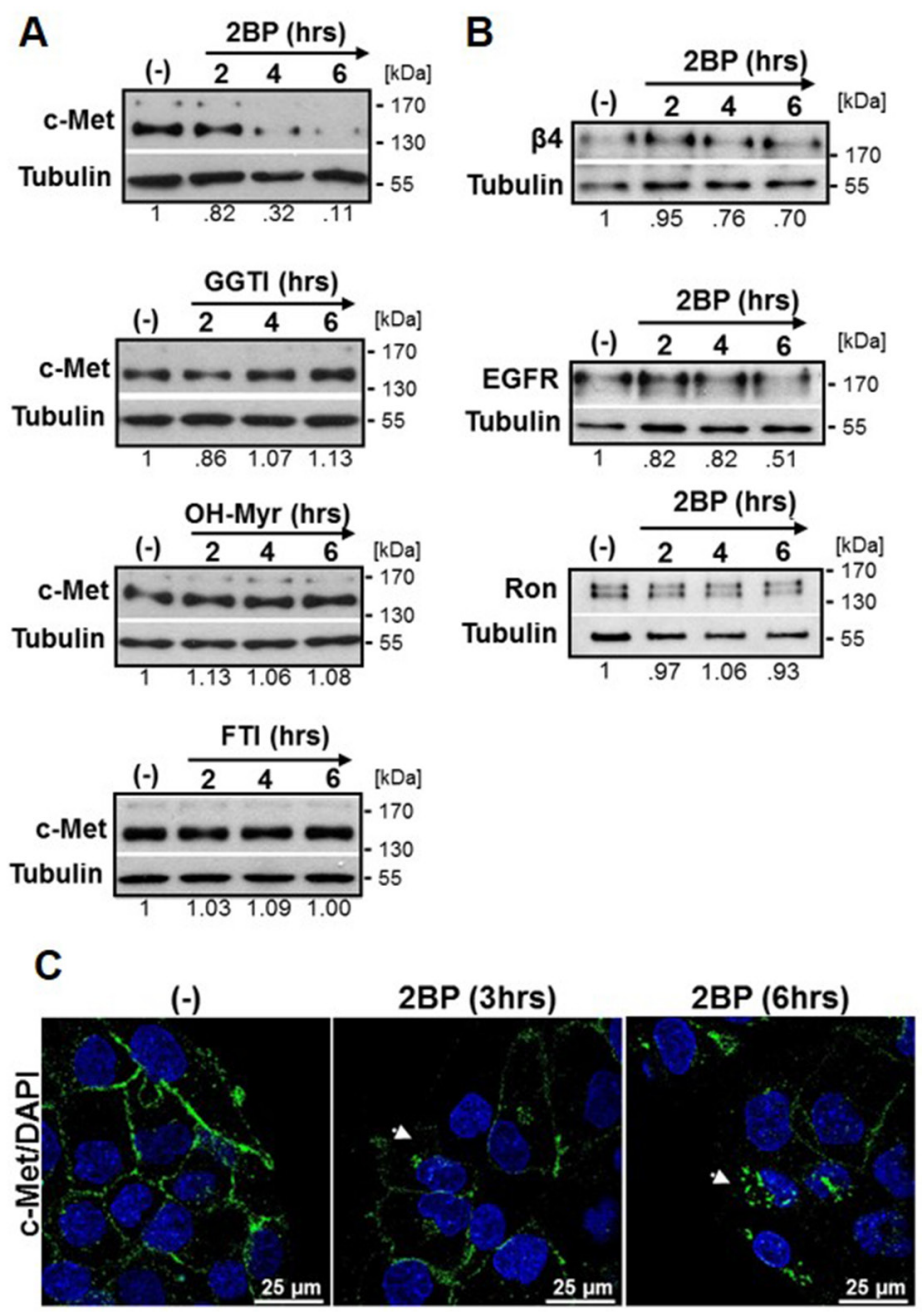
Inhibitors of palmitoylation, but not other lipid modifications, lower total c-Met protein levels **A.** DU145 prostate cancer cells were treated with DMSO (−) or 100 μM 2BP, 100 μM GGTI, 100 μM OH-Myr, or 100 μM FTI for 2, 4, or 6 hours. Western blot analysis was performed to indicate levels of **A.** c-Met or **B.** integrin β4, EGFR, or Ron. Representative blots from at least three independent experiments are shown. Densitometry indicates relative change in c-Met expression. **C.** DU145 cells were treated with 2BP for 3 or 6 hours then fixed and immunostained for c-Met (green) and DAPI (blue). Representative 60x confocal images of three independent experiments are shown. Arrows highlight perinuclear c-Met.

To determine if c-Met protein expression is stabilized by palmitoylation, we treated DU145 cells with the validated palmitoylation inhibitor 2-bromopalmitate (2BP, 100 μM) [32, 39, 40] for time points through 6 hours. Western blot analysis revealed c-Met levels were reduced over the 6 hour time course with reduction beginning at 2 hours (Figure 1A). 2BP did not inhibit FASN activity as determined by analysis of ^14^C-acetate incorporation into lipids (Supplementary Figure S1A). To determine if the effect of 2BP was specific to inhibition of palmitate attachment rather than other lipid modifications, we treated DU145 cells with inhibitors of geranylgeranylation (GGTI), myristoylation (OH-Myr), or farnesylation (FTI). These inhibitors at maximum nontoxic concentrations did not reduce the expression of c-Met over an identical time course (Figure 1A). In addition, we tested whether the effect was unique to c-Met or if a general effect was observed for other membrane spanning proteins. A 50% reduction in the epidermal growth factor receptor (EGFR) was detected at the latest time point (Figure 1B), but c-Met was reduced to a greater extent (Figure 1A). Neither integrin β4 nor the RTK Ron, was substantially reduced in response to 2BP treatment through 6 hours (Figure 1B).

In order to visualize the effect of 2BP on c-Met localization, DU145 cells were treated with 2BP for 3 or 6 hours, fluorescently labeled with antibody to c-Met, and analyzed by confocal microscopy. With increased duration of 2BP treatment, perinuclear accumulation of c-Met was apparent along with diminished plasma membrane expression, most notably at 6 hours (Figure 1C).

### Downregulation of c-Met in response to inhibition of palmitoylation occurs post-translationally

**Figure 2:**
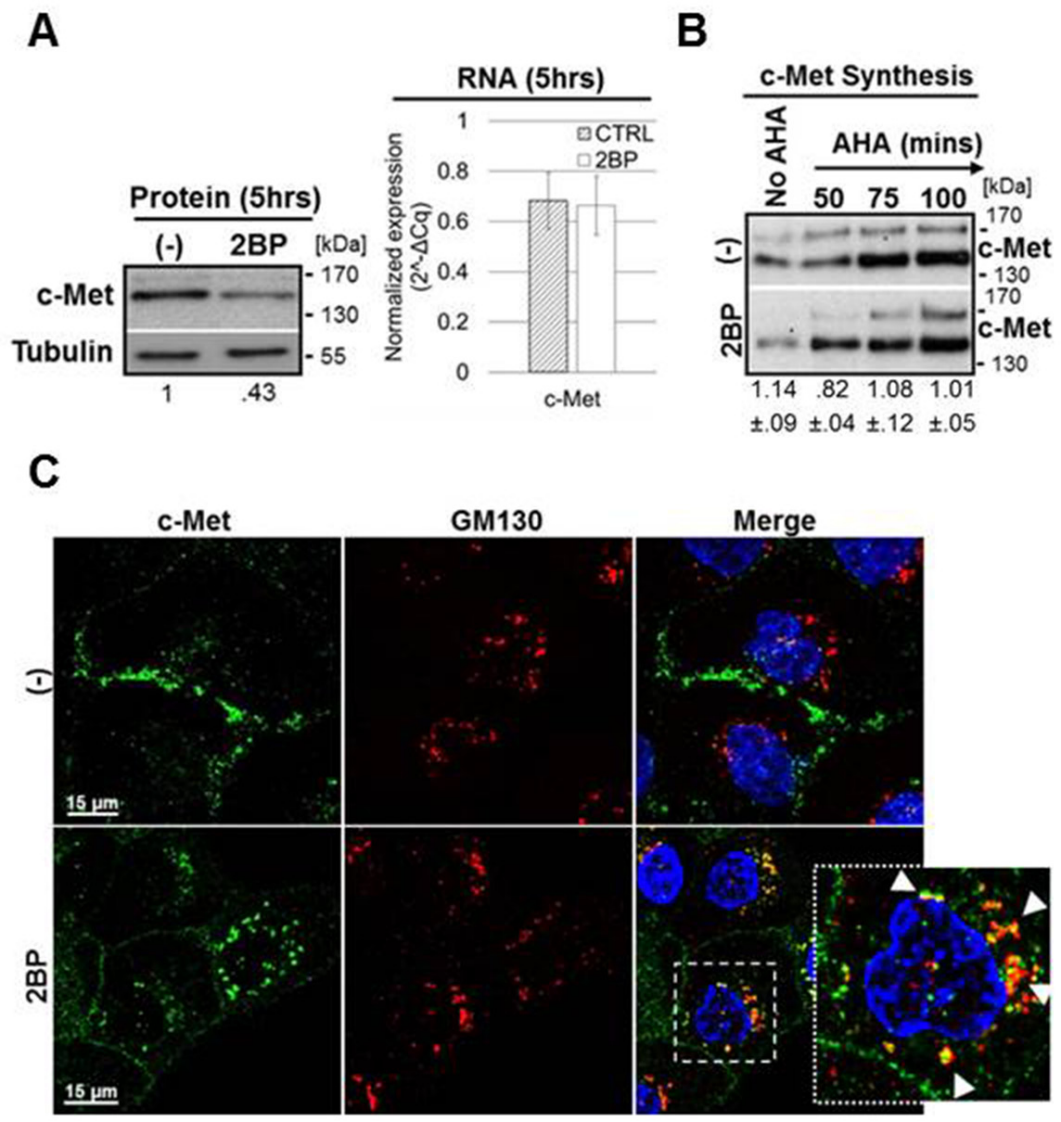
2BP treatment reduces c-Met stability post-translationally and causes a trafficking block in the Golgi **A.** DU145 cells were treated with 100 μM 2BP for 5 hours. Western blot analysis was performed to determine levels of total c-Met protein following treatment. Parallel qPCR was performed to indicate c-Met RNA levels following 2BP treatment (n=3, mean ± SEM). **B.** DU145 cells were incubated for 50, 75, or 100 minutes with azido-homoalanine (AHA) in the presence or absence of 100 μM 2BP to label newly synthesized protein with biotin through a subsequent click chemistry-based reaction. Biotinylated protein was precipitated and levels of newly synthesized c-Met were identified by western blot analysis. The representative lanes were run on the same gel, but are stacked for visual convenience ((−)/2BP densitometry ratio, n=3, mean ± SEM). **C.** DU145 cells were treated with 100 μM 2BP for 2.5 hours prior to fixing. Antibodies to c-Met (green) and the cis-Golgi marker GM130 (red) were used where indicated for immunofluorescence. Representative 60x confocal images of three independent experiments are shown. Arrows highlight areas of colocalization.

We next sought to determine whether c-Met biosynthesis or degradation was affected by 2BP treatment. Quantitative reverse-transcriptase PCR was performed on DU145 cells treated with or without 2BP for 6 hours along with parallel western blot protein analysis (Figure 2A). There was little change in the levels of c-Met mRNA concurrent with a greater than 50% reduction in c-Met protein supporting that downregulation of c-Met occurs post-transcriptionally. Rates of general protein synthesis and c-Met protein synthesis specifically were analyzed using a click-chemistry based methionine ortholog (AHA) to label newly synthesized protein. 2BP treatment did not block general protein synthesis, nor did 2BP reduce the rate of c-Met synthesis specifically (Figure 2B and Supplementary Figure S1B, S1C). Taken together, these data indicate that 2BP likely reduces the stability, and therefore half-life, of already synthesized c-Met protein.

### Inhibition of palmitoylation disrupts the trafficking of c-Met leading to reduced levels

The apparent reduced half-life of c-Met could be accounted for by an increase in the internalization and degradation of the plasma membrane c-Met pool and/or by a block in the trafficking of c-Met to the plasma membrane along the biosynthetic and secretory pathway. To distinguish these possibilities, we performed confocal microscopy on 2BP-treated DU145 cells and looked for colocalization of c-Met with markers of trafficking sites including the endoplasmic reticulum (calnexin), the *cis*-Golgi (GM130), and early endosomes (EEA1) (Figure 2C and Supplementary Figure S2). With prolonged 2BP treatment we observed accumulation of c-Met near the nucleus. There was no colocalization with early endosomes, as compared to HGF-induced internalization as control, suggesting 2BP treatment does not induce rapid internalization; however there was noticeably less c-Met at the plasma membrane (Supplementary Figure S2A). No colocalization of c-Met was detected at the ER, but there was distinct colocalization in the Golgi indicating 2BP treatment accumulated c-Met in the Golgi (Figure 2C and Supplementary Figure S2B). It is possible that inhibition of palmitoylation blocks c-Met egress from the Golgi, likely leading to c-Met degradation.

**Figure 3:**
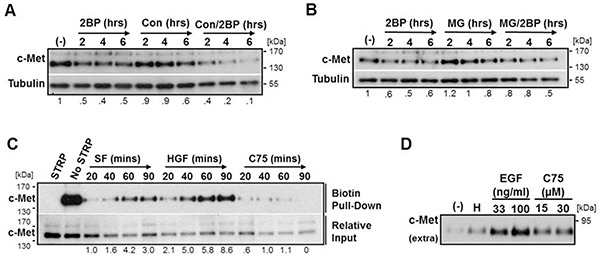
Inhibition of palmitoylation reduces c-Met levels independent of proteasomal and lysosomal degradation and increases ectodomain shedding DU145 cells were treated with an inhibitor of lysosomal acidification **A.** Concanamycin A (Con, 3 μM) or a proteasome inhibitor **B.** MG132 (MG, 5 μM) alone or in combination with 2BP (100 μM) for 2, 4, or 6 hours. **C.** Surface biotinylated DU145 cells were incubated at 37°C for the indicated time under basal serum free (SF) conditions, stimulated with HGF (33 ng/ml) to show an increased internalization rate as a positive control, or with the FASN inhibitor C75 (30 μM). All biotin remaining on the cell surface after the indicated time was stripped off (STRP). Biotinylated protein was precipitated and internalized c-Met was identified by western blot analysis. **D.** DU145 cells were treated with 33 ng/ml HGF (H), 33 or 100 ng/ml EGF, or 15 or 30 μM C75 for 8 hours. Conditioned media was collected and concentrated and western blot analysis was performed to detect the 90 kDa ectodomain of c-Met. Representative blots from three independent experiments are shown. Densitometry indicates relative change in c-Met normalized to load control.

To determine the degradation mechanism responsible for reduced c-Met levels, DU145 cells were treated with 2BP for 2, 4, or 6 hours in the presence or absence of the lysosome acidification inhibitor concanamycin A (Con) or the proteasome inhibitor MG132 (MG). Western blot analysis revealed that Con actually had an additive effect with 2BP treatment, and MG only partially prevented 2BP-induced c-Met loss (Figure 3A, 3B). To address the loss of c-Met from the plasma membrane we performed a surface biotinylation assay to monitor the kinetics of c-Met internalization. Due to constraints of 2BP toxicity in serum-free media, it was necessary to instead use the FASN inhibitor C75 to block palmitoylation, which published reports support [28–30]. Analysis of c-Met internalization revealed that inhibition of palmitoylation reduced the rate of c-Met internalization, whereas ligand stimulation as a positive control greatly increased the rate of internalization over basal levels (Figure 3C). Additionally, inhibitors of common internalization pathways were unable to prevent reduced c-Met levels in response to C75 or 2BP treatment, further supporting that an elevated rate of internalization is not involved (Supplementary Figure S3).

These experiments suggest that inhibition of palmitoylation, at least in part, causes shedding of plasma membrane associated c-Met. To test this possibility, we collected conditioned media from DU145 cells treated with either HGF or EGF as positive controls or C75 to inhibit palmitoylation to detect possible increases in the previously published 90 kDa shed c-Met ectodomain fragment [7]. Using an antibody to an extracellular epitope of c-Met, it was determined that, in fact, there was a consistent increase in the amount of shed 90 kDa c-Met fragment upon inhibition of FASN with C75 (Figure 3D).

### c-Met is S-palmitoylated

**Figure 4:**
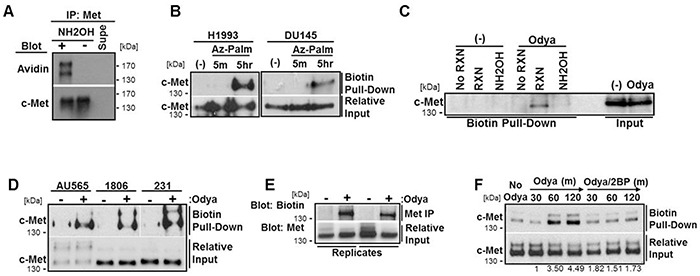
c-Met is palmitoylated via a hydroxylamine-sensitive thioester bond **A.** An acyl-biotin exchange technique involving the blocking of free cysteine residues, removal of palmitate from cysteine by hydroxylamine (NH_2_OH), and biotin linkage to these cysteines, was performed on lysates from DU145 cells. Western blot analysis was used to detect biotinylated c-Met. **B.** H1993 or DU145 cells were incubated with azide-linked palmitate (Az-Palm) for indicated time to label palmitoylated proteins. Biotin was linked to palmitoylated protein with the click chemistry reaction. **C.** DU145 cells were incubated for 5 hours with (Odya) or without (No Odya) palmitate ortholog. Samples were divided and either not biotinylated through the click reaction (No RXN), reduced prior to the reaction (NH_2_OH), or biotinylated and not reduced (RXN). **D.** Breast cancer cell lines MDA-MB-231, HCC1806, and AU565 were incubated with/without Odya for 5 hours. For each click reaction experiment, western blot analysis for c-Met was performed following biotin pull-down. Gap indicates cropped blot. Aligned input was run separately. **E.** H1994 cells were incubated with Odya for 5 hours. c-Met was immunoprecipitated and processed through the click reaction. Samples were probed for biotin with streptavidin-HRP by western blot. Two replicate samples are shown along with relative IP input for c-Met. **F.** H1993 cells were incubated with Odya in the presence or absence of 100 μM 2BP for 30, 60, or 120 mins. Click reaction was performed followed by biotin pull-down and western blot analysis for c-Met. Representative blots from at least three independent experiments are shown.

To test whether c-Met is palmitoylated, we initially utilized an acyl-biotin exchange technique for labeling cysteine residues modified via a thioester bond, like S-palmitoylation. In brief, thioester-linked acyl groups were exchanged with biotin on immunoprecipitated c-Met protein in a hydroxylamine-dependent reaction (NH_2_OH) [37]. These samples from DU145 cells were separated by SDS-PAGE and blotted with streptavidin-HRP to detect the presence of biotinylated c-Met. Western blot analysis indicated that the initial covalent block of unbound cysteine residues was complete (−NH_2_OH) and that c-Met was biotinylated only when acyl-modified cysteines were reduced and exposed to biotin binding (+NH_2_OH) (Figure 4A).

Next we used click chemistry-based metabolic labeling with palmitate orthologs azide-palmitate (Az-Palm) or alkyne-linked 17-Octadecynoic acid (Odya) which are covalently cross-linkable with biotin [41–43]. After incubating cells with palmitate orthologs, lysates were processed through the click reaction, biotinylated proteins were pulled-down, and samples were immunoblotted for c-Met. Ortholog labeling of c-Met was observed in every cell line assessed including the H1993 lung cancer cell line as well as AU565, HCC1806, and MDA-MB-231 breast cancer cell lines (Figure 4B, 4D). c-Met labeling was equally detectable using both palmitate orthologs (Az-Palm and Odya, Figure 4B, 4D) and exhibited time dependence (5 min vs 5 hrs, Figure 4B), supporting the technique's validity. In Figure 4C, DU145 cells were treated with 200 μM Odya or vehicle (−) for 4 hours to allow incorporation onto palmitoylated proteins. To test reaction specificity, equal protein from each sample was divided into those processed through the click reaction (RXN) versus those not (No RXN) and samples that were processed after reducing thioester bonds with NH_2_OH thereby removing any cysteine-linked Odya. Western blotting revealed c-Met pull-down was dependent on the click-reaction and was prevented by reducing agent, again supporting S-palmitoylation (Figure 4C).

The H1993 cell line with amplified c-Met, compared to the relatively low levels in DU145 cells, was used for several experiments hampered by limited c-Met levels. To test the reverse approach of detection, c-Met was immunoprecipitated from Odya-incubated H1993 cell lysates. Isolated protein was reduced with tris(2-carboxyethyl)phosphine (TCEP) to break disulfide bonds while maintaining the thioester-linked Odya and then click processed. Probing samples with streptavidin-HRP following SDS-PAGE again revealed Odya-incorporated c-Met (Figure 4E). To determine if 2BP treatment inhibited the palmitoylation of c-Met, we incubated H1993 lung cancer cells with Odya over time in the presence or absence of 100 μM 2BP. Following processing and pull-down, western blot analysis revealed that c-Met incorporated Odya over time and that 2BP treatment prevented this palmitoylation (Figure 4F).

**Figure 5:**
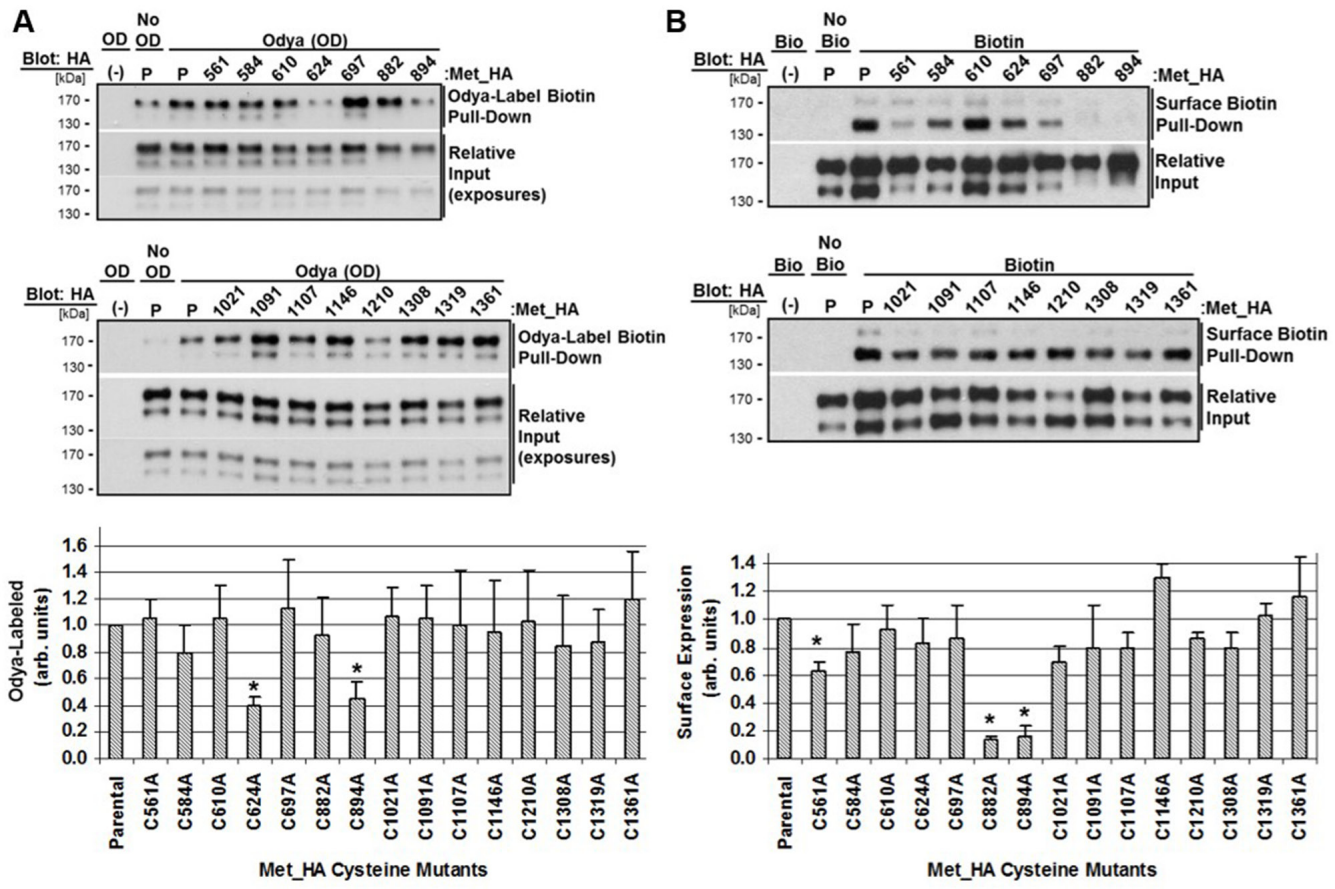
Characterization of c-Met cysteine mutant palmitoylation and surface expression HEK293 cells were transfected with parental Met_HA (P) or the indicated cysteine mutant Met_HA plasmid for 24 hours. **A.** Odya-labeling was performed (OD) or not (No OD) for 2 hours followed by biotin-pulldown and western blotting for HA. **B.** Cell surface biotinylation at 4°C was performed (Bio) or not (No Bio) followed by biotin-pull-down and western blotting for HA. Representative blots are shown with dual input exposures for clarity. Quantitation of replicate densitometry is graphed for each mutant in at least three independent experiments. *P<0.05, significant differences compared to parental values set to 1 (n=3, mean ± SEM). Differences in Odya-labeling were relative to 170 kDa precursor band, and surface expression was relative to the140 kDa mature band.

To determine the site of acyl-group linkage we engineered fifteen individual HA-tagged cysteine (C/A) mutants representing all cysteines throughout the β-chain not previously shown to be involved in disulfide bonds, except C561 as a control [44]. C/A mutants were transfected into HEK293 cells overnight followed by 5 hour incubation with Odya. Odya-labeled protein was isolated by biotin pull-down and probed for HA. Western blot analysis and quantitation of replicate densitometry (170 kDa band) comparing Odya labeling of the parental c-Met to C/A mutants revealed C624A and C894A were consistently Odya-labeled to a lesser extent. C882A and C894A had a clear defect in processing as the 140 kDa mature form was weakly detected and migrated slower. This processing defect wouldn't necessarily prevent palmitoylation of the precursor prior to proteolytic maturation (Figure 5A).

**Figure 6:**
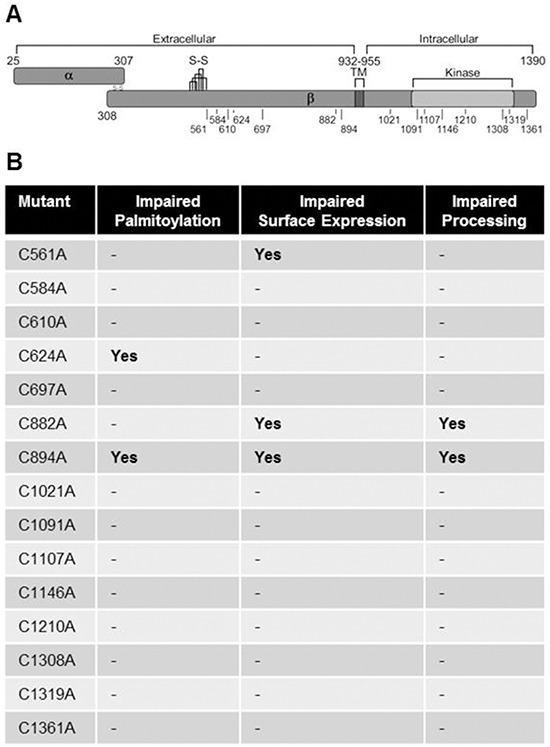
c-Met cysteine mutation sites **A.** The c-Met protein is synthesized as a 170 kDa single chain precursor that is cleaved in the Golgi into a disulfide-linked α chain (50 kDa) and β chain (140 kDa). C/A mutations were made for each cysteine residue throughout the β chain excluding those within the cysteine-rich plexin domain (S-S) except one (C561). The diagram indicates the location of each mutationwith respect to the transmembrane domain (TM), kinase domain, and intracellular/extracellular regions. **B.** The table summarizes findings with regards to Odya-labeling, surface biotinylation, and observed processing of the 170 kDa full-length into the 140 kDa β chain.

To determine which cysteine residue/s were required for trafficking of c-Met to the plasma membrane, we performed surface biotinylation after transfection with parental c-Met or C/A mutants. Surface expressed biotinylated protein was pulled-down and probed for HA. Western blot analysis and quantitation of replicate densitometry (140 kDa band) revealed most mutants trafficked to the plasma membrane comparable to parental c-Met except C882A and C894A, which aren't efficiently processed to the mature form, and control C561A which was expressed at the surface 40% less (Figure 5B). Results from the palmitoylation and surface biotinylation assays are summarized in Figure 6 along with a schematic representation of the site of each mutation (Figure 6A, 6B).

### c-Met palmitoylation occurs in the ER

Based on the compelling data that c-Met is palmitoylated, we investigated the dynamics of palmitoylation during the lifespan of c-Met. Early studies on the biosynthesis of c-Met determined that the protein was synthesized as a 170 kDa precursor that is subsequently cleaved in the Golgi to α- and β-chains that are linked via disulfide bonds. Under reducing conditions, the mature β-chain migrates at 140 kDa by SDS-PAGE [2].

**Figure 7:**
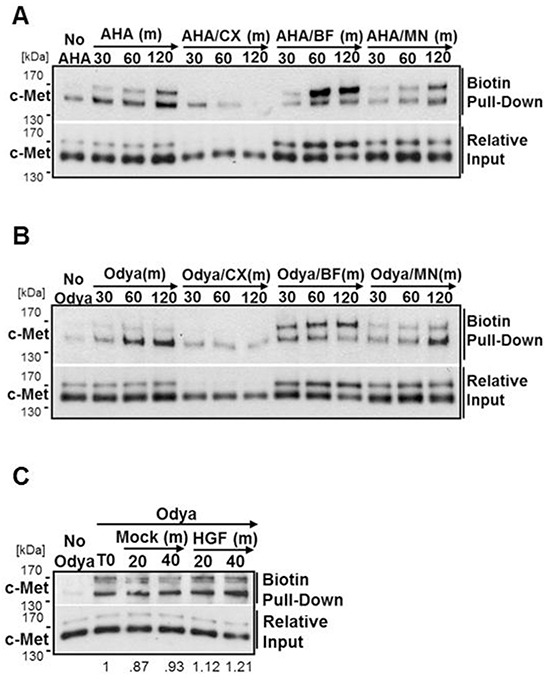
c-Met is stably palmitoylated in the ER H1993 cells were incubated with azido-homoalanine (AHA) **A.** or Odya **B.** alone or with cycloheximide (CX, 10 μg/ml), brefeldin A (BF, 2 μM), or monensin (MN, 2 μM) for 30, 60, or 120 mins (m). **C.** DU145 cells were incubated with Odya for 2 hours (T0) before chase periods of 20 or 40 minutes in the presence of Odya +/− HGF. For each, the click reaction was performed followed by biotin pull-down and western blot analysis to detect the presence of c-Met. Representative blots from three independent experiments are shown.

To ensure accurate experimental disruption of c-Met biosynthesis, H1993 cells were first incubated with AHA, to monitor nascent protein, in the presence or absence of cycloheximide (CX, 10 μg/ml) to inhibit protein synthesis, brefeldin A (BF, 2 μM) to inhibit protein transport from the ER to the *cis*-Golgi, or monensin (MN, 2 μM) to prevent transport out of the Golgi network to the plasma membrane. When incubated with AHA for time points within 2 hours in combination with BF, the 170 kDa c-Met precursor accumulated having not entered the Golgi for precursor cleavage. Furthermore, CX treatment effectively prevented new synthesis of c-Met, and MN did not disrupt synthesis or cleavage of c-Met inside the Golgi (Figure 7A). Moreover, we found that the antibody to c-Met used for immunofluorescence does not detect c-Met in its uncleaved precursor form. Exploiting this, DU145 cells were treated with reversible BF or MN and immunostained to observe c-Met distribution. Perinuclear c-Met is not detected under BF treatment until the compound is washed away allowing a bolus of c-Met to emerge processed in the Golgi, whereas MN treatment causes perinuclear accumulation of processed c-Met (Supplementary Figure S4). Prolonged treatment with either inhibitor leads to loss of c-Met staining from the plasma membrane due to basal internalization and degradation, similar to what is observed with 2BP treatment (Supplementary Figure S4).

With this information, we sought to determine the location and kinetics of c-Met palmitoylation. H1993 cells were labeled with Odya for time points within 2 hours in the presence or absence of CX, BF, or MN and western blot analysis was performed to observe palmitoylated c-Met. In the presence of CX treatment there is no detectable level of c-Met palmitoylation above the pull-down background (No Odya) (Figure 7B). Treatment with BF revealed the 170 kDa precursor form of c-Met is palmitoylated and that c-Met palmitoylation must therefore occur in the ER. MN treatment does not appear to impede the kinetics of c-Met palmitoylation under basal growth conditions, consistent with palmitoylation occurring in the ER (Figure 7B). As detailed with other proteins, it is possible that the dynamics of c-Met palmitoylation are altered in response to its ligand, HGF, as it is activated and internalized. To test this, we incubated DU145 cells for 2 hours (T0) with Odya to accumulate biotinylated c-Met at the plasma membrane. Without changing the media, cells were chased for additional 20 or 40 minutes in the presence or absence of HGF. As indicated by western blot analysis, HGF does not cause a noticeable increase or decrease in the amount of palmitoylated c-Met (Figure 7C).

## DISCUSSION

This report is the first evidence that the RTK c-Met is palmitoylated, and that inhibition of palmitoylation reduces c-Met levels. Our data highlight the sensitivity of c-Met to inhibition of palmitoylation as compared to other lipid modifications. c-Met was also unique relative to other transmembrane proteins including integrin β4 and Ron, although EGFR levels were consistently reduced at longer time points. A recent report suggests FASN-dependent palmitoylation can elevate the activity and levels of a sub-population of EGFR [45]. There is no evidence for palmitoylation of the Met family member Ron; and palmitoylation of integrin β4 is known to heighten its activity but not necessarily expression levels [32].

Real-Time PCR and metabolic labeling of nascent protein indicated that c-Met was reduced post-translationally. c-Met expression diminished from the cell surface and accumulated in a perinuclear compartment colocalized with a *cis*-Golgi marker, suggesting c-Met is blocked along the biosynthetic route and possibly rerouted for degradation upon build-up. In accordance with these results, 2BP does not completely deplete c-Met protein but leads to a greatly reduced steady-state. Marked reduction of c-Met at the plasma membrane could be due to internalization or ectodomain shedding. Surface biotinylation assays indicated c-Met internalization was greatly reduced in response to FASN inhibition compared to basal or HGF-stimulated rates. In addition, we detected elevated levels of the 90 kDa c-Met ectodomain in media following treatment with a FASN inhibitor, suggesting inhibition of palmitoylation causes a loss of c-Met surface expression, at least in part, through ectodomain shedding. Blocking the proteasomal or lysosomal degradation pathways individually was unable to greatly prevent 2BP-induced c-Met loss. Multiple reports detail the paradoxical effects these degradation pathways have on c-Met trafficking and stability [46–48]. We find it likely that distinct intracellular pools of c-Met are degraded through different, possibly compensatory, pathways. Regardless, inhibition of palmitoylation prevents nascent c-Met protein from reaching the plasma membrane.

We next determined that c-Met itself could be S-palmitoylated using an acyl-biotin exchange technique which has been used extensively to identify proteins with thioester-linked modifications. To support these data we used biorthogonal palmitate to biotin-label palmitoylated proteins following click chemistry. This relatively new technique allows for a more rapid and tractable study of palmitoylated proteins, compared to radiolabeling, with validated specificity [49–51]. c-Met was determined to be palmitoylated in every cell line tested, with two different bioorthogonal reporters (azide and alkyne), and in a manner sensitive to reducing agent which signifies cysteine-linked S-palmitoylation. Palmitoylation of c-Met was a time-dependent reaction excluding nonspecific post-lysis enzymatic activity. To our knowledge, this is the first evidence of c-Met being palmitoylated in any cell type.

To elucidate the dynamics of c-Met palmitoylation, we used inhibitors to block steps of c-Met biosynthesis. CX treatment completely blocked pulse labeling of c-Met and BF treatment greatly reduced detection of palmitoylated mature c-Met. We find it convincing that c-Met is palmitoylated prior to leaving the ER in its precleaved 170 kDa precursor structure. In this model, palmitate would be attached to precursor protein in the ER, likely catalyzed by a resident acyltransferase. Palmitate would remain attached to one or more cysteine residue/s within the β-chain through cleavage in the Golgi and as it is trafficked to the plasma membrane. Given that neither MN treatment nor HGF stimulation greatly affected the amount of c-Met palmitoylated over time, we predict that neither association with the plasma membrane nor internalization and recycling, control or are controlled by, dynamic palmitoylation. Rather, this is a stable attachment that remains throughout the duration of the molecule's life. Given these findings, it seems probable that the induction of c-Met shedding in response to inhibition of palmitoylation is an indirect effect – the perturbation of proteins that control the shedding process.

Blocking palmitoylation did not prevent exit from the ER or cleavage in the Golgi, but it did impede exit of c-Met out of the Golgi through the secretory pathway. It is possible that the requirement of palmitoylation for c-Met to exit the Golgi is related to trans-Golgi lipid raft structures [52–54]. In the absence of palmitoylation, c-Met may not integrate into these membrane domains, potentially required for Golgi exit. Accumulation would likely result in eventual degradation by a misfolding-like mechanism. Future work will be required to test this hypothesis.

Multiple methods were employed to identify the cysteine residue/s required for c-Met palmitoylation. Technical issues impeded complete consistent sequence coverage from mass spectrometry, and the labile nature of the lipid attachment made detection unreliable. Site-directed mutagenesis of all β-chain cysteine residues not involved in intramolecular disulfide bonds, except C561, revealed two sites that reduced Odya labeling of c-Met. C894 and C624 are evolutionarily conserved residues N-terminal to the transmembrane domain (aa933-955) residing extracellular at the plasma membrane. Importantly, C894A exhibits a clear defect in proteolytic processing as indicated by altered PAGE migration; however, given palmitoylation occurs before cleavage, the results still suggest this cysteine, along with C624, are probable palmitoylation sites.

Type-1 membrane proteins are most commonly palmitoylated on cytosolic-facing cysteine residues; however, our evidence supports lumenal palmitoylation of c-Met in the ER resulting in an extracellular palmitoylated residue. Of the 21 DHHC family palmitoyl acyltransferases, DHHC12, 13, 14, and 16 are known to reside at the ER and were detected by PCR in the cell lines used to demonstrate c-Met palmitoylation (data not shown)[55, 56]. Although not unprecedented, lumenal palmitoylation is less mechanistically obvious. This mechanism would require lumenal orientation of the DHHC active site at the ER, if only transiently; as well as transport of palmitoyl-CoA into the lumen, or activation of palmitate within the lumen. Published data supports the occurrence of both [57–60] and lumenal palmitoylation has been documented for proteins including apolipoprotein-B and amyloid precursor protein [61–63].

A parallel surface biotinylation experiment was performed to determine whether a direct relationship could be observed between impaired palmitoylation and trafficking of c-Met to the cell surface. As expected, C894A and C882A were not expressed at the plasma membrane because of the processing impairment. C561A was also expressed at the plasma membrane less efficiently; however, C624, which is palmitoylation impaired, seems to reach the plasma membrane equivalently.

Although C624A and C894A greatly reduced palmitoylation, considering no single cysteine mutation completely ablated palmitoylation, surface expression or c-Met levels (albeit CMV promoter-driven expression artificially amplifies levels), we find it likely that multiple cysteine residues may be palmitoylated and required for proper trafficking of c-Met. Multi-site palmitoylation has been observed with integrin β4 and numerous other palmitoylated proteins [64]. Future studies will delineate the consequence and pattern of c-Met palmitoylation with greater resolution.

These findings reveal a novel post-translational modification that is necessary for the trafficking and stability of a clinically important receptor tyrosine kinase. There is abundant evidence implicating c-Met as a molecular target for cancer therapy, and its biology is important to embryonic development, liver regeneration, and fibrosis. Greater understanding of the factors regulating c-Met biosynthesis, trafficking, and activity is essential for future design of optimized therapeutic intervention. Exploiting the need for lipidation may serve as such a therapeutic modality.

## MATERIALS AND METHODS

### Cell culture and reagents

All cell lines used were obtained from ATCC (Manassas, VA) and have since been validated (Promega). DU145 (prostate carcinoma), H1993 (lung carcinoma), AU565, and HCC1806 (breast carcinoma) cells were maintained in RPMI 1640 media (Cellgro, Herndon, VA), MDA-MB-231 (breast carcinoma) cells were maintained in DMEM (Cellgro), and HEK293 cells in EMEM (Cellgro); each containing 10% fetal bovine serum (FBS) (Gemini, West Sacramento, CA). Cells were kept at 37°C with 5.0% CO_2_. Monensin and brefeldin A were obtained from Biolegend (San Diego, CA), Y27637 from Stem Cell Technology (Vancouver, BC), C75, 2-hydroxymyristic acid, and 17-Octadecynoic Acid from Caymen Chemicals (Ann Arbor, MI), cycloheximide, recombinant EGF, geranylgeranyltransferase inhibitor, farnesyltransferase inhibitor, vinblastine, latrunculin, dansylcadaverine, ethylisopropyl amiloride, methyl-β-cyclodextrin, nystatin, and 2-bromopalmitate were obtained from Sigma-Aldrich (St. Louis, MO). Click-iT® reaction buffers, azido-homoalanine, azide-palmitate, and methionine-free RPMI, were obtained from Invitrogen (Carlsbad, CA). Recombinant HGF was obtained from EMD Millipore (Darmstadt, Germany). Streptavidin Sepharose High Performance beads were obtained from GE Healthcare (Pittsburgh, PA).

### Western blotting

Cells were seeded to 70% confluency in 24-well plates before treatments. Inhibitors were spiked into the media and incubated for the indicated times prior to cell lysis. Lysates were taken in boiling Laemmli buffer (125 mM Tris-HCl, 4% SDS, 0.01% bromophenol blue, 30% sucrose) with 0.05% β-mercaptoethanol (BME) and boiled for 5 minutes. Samples were analyzed by SDS-PAGE and blotted with the indicated primary antibodies: c-Met CVD13 (1:1000)(Invitrogen), integrin β4 H-101 (1:1000), Ron-β C-20 (1:750), and EGFR 1005 (1:750)(Santa Cruz Biotechnology, Santa Cruz, CA,), and β-tubulin (Neomarkers, Fremont, CA, 1:5000). High sensitivity streptavidin-HRP was obtained from Pierce Thermo Scientific (Rockford, IL, 1:10000). Blots were subsequently probed with horseradish peroxidase-conjugated secondary antibodies (Amersham Biosciences, Pittsburgh, PA), and detection was acquired with ECL (Amersham Biosciences).

### Quantitative reverse transcriptase PCR

DU145 cells were seeded to 70% confluency in 10 cm dishes prior to treatment in serum-free media with or without 100 μM 2BP for 5 hours. Following cell collection and centrifugation, cells were resuspended in Trizol (Invitrogen) and RNA was isolated according to manufacturer instructions. The SuperScript First-Strand kit (Life Technologies; Grand Island, NY) was used for cDNA synthesis. RT^2^ SYBR Green Flour FAST Mastermix (Qiagen; Valencia, CA) was used in qPCR reactions. Cycling conditions were 95°C 10 minutes, followed by 40 cycles of 95°C 10 seconds and 55°C 30 seconds. Melt curves were ran each time and reverse transcriptase negative and template negative controls were used. Reactions were carried out using a Bio-Rad CFX96 Real-Time PCR Detection System with Bio-Rad CFX Manager 3.0 software. Primers were designed using Integrated DNA Technologies (Coralville, IA) PrimerQuest software and sequences used include (5′ to 3′): Met forward: ACCGAAAGATAAACCTCTCATA, Met reverse: TGCTAGTGCCTCTTTACAC, GAPDH forward: GTCGGAGTCAACGGATTT, GAPDH reverse: AGTTGAGGTCAATGAAGGG.

### FASN activity assay

To label newly synthesized fatty acid, cells were pulsed with 1 μCi ^14^C-acetate (GE Healthcare, Piscataway, NJ) for 2 hours, washed with phosphate buffered saline (PBS), and lysed in hypotonic buffer (1 mM dithiothreitol (DTT), 1 mM ethylenediaminetetraacetic acid (EDTA), 20 mM Tris-HCl, pH 7.5). Lipids were extracted with chloroform:methanol (2:1 v/v) and ^14^C-acetate incorporation was quantified by scintillation counting.

### Confocal microscopy

Cells were grown to 50% confluency on glass coverslips in a 6-well plate. Following treatments cells were fixed in 4% PFA, washed twice with PBS, and blocked for 30 minutes in 10% donkey serum PBS with 0.1% saponin (DSP). Primary antibodies c-Met (1:70) (R&D Systems), GM130 (1:100), EEA1 (1:100), or calnexin (1:100) (BD Biosciences) were added simultaneously in DSP for 2 hours at room temperature. After washing three times with PBS, secondary antibodies (Jackson Immunoresearch) were added in DSP for 2 hours at room temperature. Coverslips were washed again three times with PBS and then mounted on coverslips with DAPI slowfade (Invitrogen). Representative images were taken on a Leica TCS SP5 Confocal Microscope at 60x magnification with oil immersion. Representative images are shown as enhanced using ImageJ software.

### Click chemistry palmitoylation assays

Cells were grown to 80% confluency in 10 cm dishes. All dishes were pretreated with growth media containing fatty acid-free 10% FBS (Gemini) with vehicle or treatment for indicated times. Media is replaced with fresh fatty acid-free growth media containing alkyne-labeled (17-Odya, Cayman) palmitic acid or azide-labeled (Az-Palm, Invitrogen) that was sonicated for 5 minutes to improve solubility with or without treatments. Cells were incubated for 4 hours to allow incorporation of palmitate orthologs. Cell lysates were taken in NP-40 lysis buffer (25mM Tris-HCl, pH 7.4, 150mM NaCl, 1mM EDTA, 1% NP-40 and 5% glycerol) containing protease inhibitor cocktail (Roche) at 4°C. Lysates were processed by 30 minutes of end-over-end rotation at 4°C followed by removal of debris by centrifugation at 13,000xg for 5 minutes. Protein concentrations were determined and 200 μg of each sample were methanol-chloroform precipitated. Precipitated protein was resuspended in 50 μl of 1% SDS reaction buffer (50 mM Tris-HCl, 1% SDS, pH 8.0) and the click chemistry reaction was performed according to the manufacturer's protocol using the Click-iT® reaction buffer kit and corresponding biotin-based detection reagent (Invitrogen). Following linkage of the palmitate ortholog to biotin, protein was again precipitated and resuspended in 100 μl of 1% SDS. Once protein was solubilized, SDS was diluted out with 1 ml of NP-40 lysis buffer. Samples were precleared for 1 hour at 4°C with A/G agarose beads (Santa Cruz) and centrifuged at 13,000xg for 5 minutes. After preclear, 100 μl of prewashed streptavidin-conjugated sepharose beads (GE Healthcare) were added to each supernatant. Samples were rotated end-over-end overnight at 4°C. IP samples were washed 5 times (50 volumes) in NP-40 lysis buffer, before protein was eluted with 30 μl of Laemmli buffer with BME. Samples were analyzed by western blot using antibody to c-Met (Invitrogen).

### Click chemistry nascent protein detection

Cell lines were grown to 70% confluency in 10 cm dishes. Cells were incubated in methionine-free growth media (Invitrogen) containing 10% FBS for 1 hour prior to treatments. Any pretreatment inhibitor was spiked into the well for the last 30 minutes of the methionine-starve period. Media was then replaced with fresh methionine-free growth media containing 10% FBS with or without 30 μM azido-homoalanine (AHA) (Invitrogen) to label nascent proteins with or without indicated treatments. Processing of lysates, click-chemistry reaction, and pull-down of biotinylated protein was performed as detailed above.

### Surface biotinylation

Cells were grown to 70% confluency in 10 cm dishes. Cells were washed once with 4°C PBS to prevent membrane internalization. Surface-exposed proteins were biotinylated with 0.1 mg/ml EZ-Link biotin (Invitrogen) in PBS at 4°C for 15 minutes. Excess biotin was quenched and removed with three 5-minute washes of 100 mM glycine in PBS at 4°C. All plates, except one complete strip control, were then incubated with 37°C prewarmed serum-free media containing the indicated treatments for chase periods to allow internalization. After indicated chase periods, cell were washed twice with 4°C PBS to halt the experiment. Remaining extracellular biotin was removed, from all but a no strip control, with MESNA buffer in PBS (100 mM MESNA, 100 mM NaCl, 1 mM EDTA, 50 mM Tris base, 0.2% BSA, pH 8.6) for three 5-minute washes at 4°C. Residual MESNA was removed with six washes with 4°C PBS. Cells lysates were taken in NP-40 lysis buffer containing protease inhibitor cocktail (Roche) at 4°C. Processing of lysates and precipitation of biotinylated protein from equal protein samples was performed as detailed above. Equal protein was analyzed for c-Met by western blotting to indicate amount of c-Met internalized over time.

### Receptor shedding

Cells were grown to 60% confluency in 10 cm dishes. Cells were washed twice with room temperature PBS and then treated with 3.5 ml serum free media containing the indicated treatments (EGF 50, 100 ng/ml; HGF 33 ng/ml; C75 10, 20 μM) and incubated for the indicated time. Conditioned media was collected and washed. Cells were lysed in NP-40 lysis buffer. Conditioned media was centrifuged at 2100 rpm for five minutes at 4°C to pellet cell debris. Supernatant was transferred to a clean tube for TCA precipitation. Fresh 2% NaDeoxycholate was added 1:100, gently mixed and incubated at room temperature for 15 minutes. Fresh TCA was then added at 1:10, vortexed, and incubated at room temperature for 1 hour. The protein was centrifuged out at 5500 rpm for 10 minutes at 4°C. The supernatant was aspirated and the pellet was allowed to dry. The pellet was then washed in 200 μl ice cold acetone, vortexed, and left on ice for an additional 15 minutes, followed by a repeat centrifugation at 5500 rpm for 10 minutes at 4°C. Supernatants were then aspirated and pellets dried. 100 μl of Laemmli buffer with BME was added to each protein pellet. Equal volumes were loaded and analyzed by SDS-PAGE blotting with the DL-21 antibody to an extracellular c-Met epitope [7].

### Mutagenesis and transfection

c-Met isoform b (NM_00236.2) was restriction digested from pLenti-MetGFP (Addgene: 37560, Dr. David Rimm) and ligated into the multi-cloning site of pCMV6-AC-HA (OriGene: PS100004) giving a c-terminal HA-tag. Fifteen separate site-directed mutants were engineered (C/A: 561, 584, 610, 624, 697, 882, 894, 1021, 1091, 1107, 1146, 1210, 1308, 1319, and 1361) using Agilent QuickChange II XL reagents (La Jolla, CA). Each mutant was verified by sequencing (Macrogen, Baltimore, MD). Transfections were performed using Continuum reagent (Gemini) according to manufacturer instructions.

### Statistical analysis

Data are expressed as means ± SEM. Statistical analysis was performed using Student's *t*-test (two tailed) with *P*-value <0.05 as significant.

## SUPPLEMENTARY FIGURES


