# Supplementary material for: Palmitoylation regulates the intracellular trafficking and stability of c-Met
Source: Oncotarget. 2016 Apr 12;7(22):32664–77. doi: 10.18632/oncotarget.8706 (PMC5078042; doi:10.18632/oncotarget.8706)
Supplement: Supplementary file 1 [file oncotarget-07-32664-s001.pdf]

Palmitoylation regulates the intracellular trafficking and stability of c-Met

SUPPLEMENTARY FIGURES

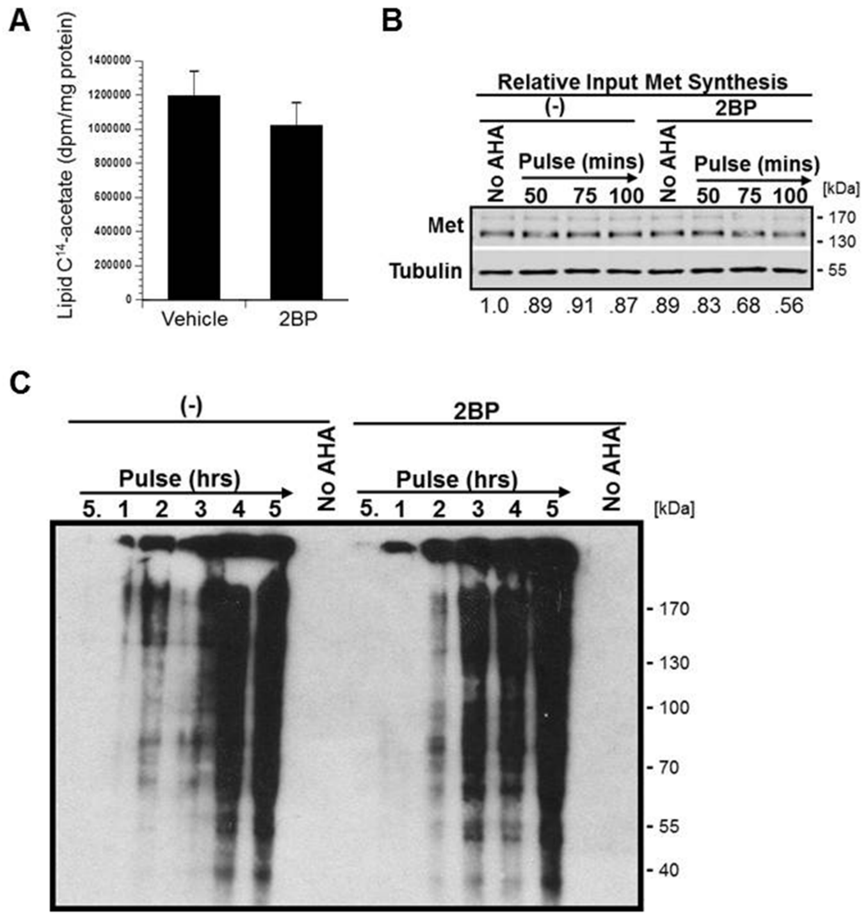

Supplementary Figure S1: 2BP treatment does not inhibit FASN or slow c-Met synthesis. A. DU145 cells were treated with 100 µM 2BP for 2 hours prior to the addition of ^{14}C-acetate for 2 hours. **B.** DU145 cells were pulsed for 50, 75, or 100 minutes with azido-homoalanine (AHA) in the presence or absence of 100 µM 2BP. Relative levels of total cell lysate c-Met protein were indicated under each condition by western blot analysis. **C.** DU145 cells were pulsed for .5, 1, 2, 3, 4, or 5 hours with AHA in the presence or absence of 100 µM 2BP to label newly synthesized protein with biotin through a subsequent click chemistry-based reaction. Total cell lysate biotinylated protein was analyzed by western blotting with Streptavidin-HRP. Representative blots are shown.

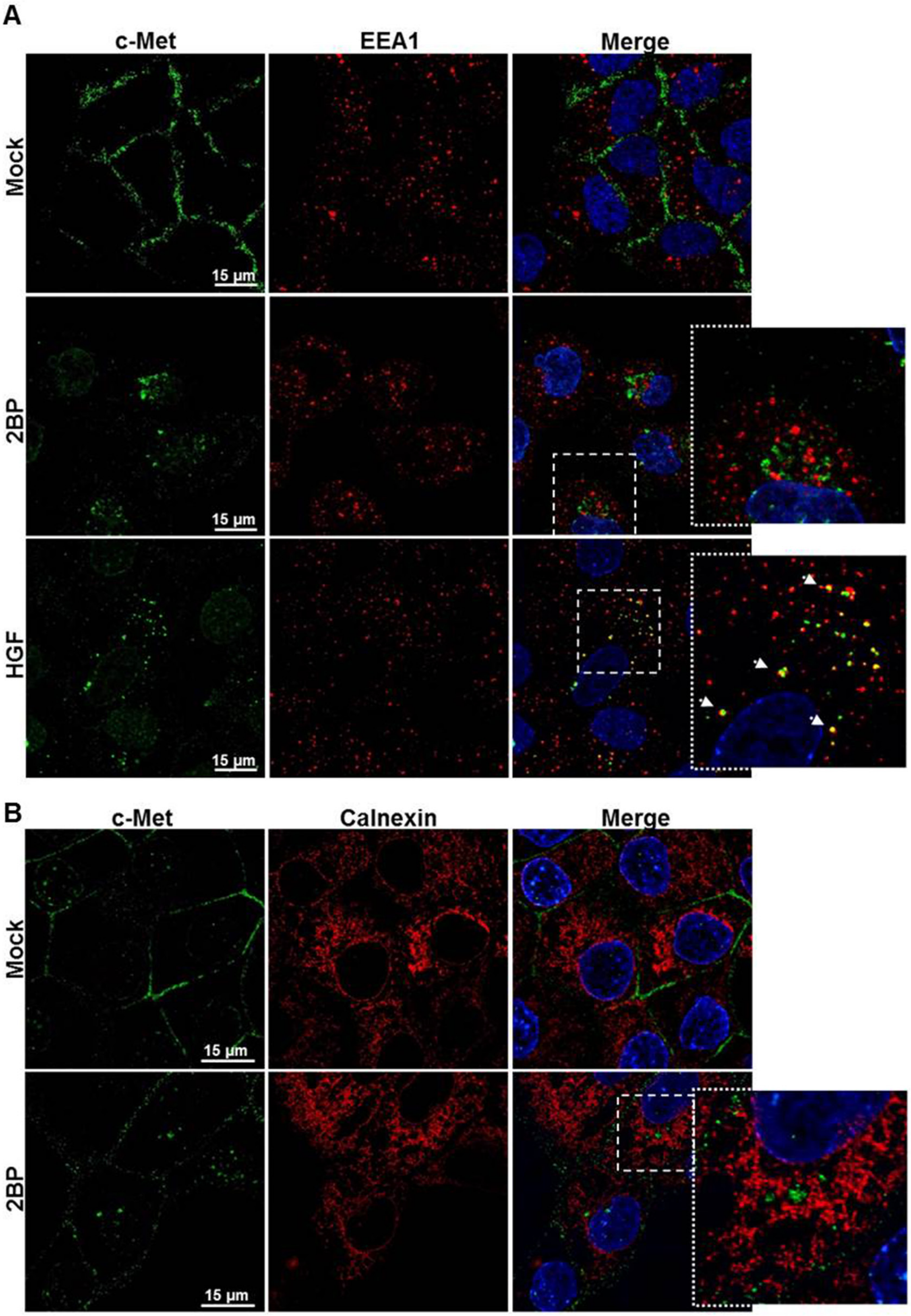

Supplementary Figure S2: c-Met does not accumulate in early endosomes or the endoplasmic reticulum during 2BP treatment. DU145 cells were treated with or without 100 μM 2BP for 2.5 hours or HGF for 1 hour prior to fixing. Antibodies to c-Met (green) or the **A.** Early Endosome marker EEA1 (red) or **B.** ER marker calnexin (red) were used where indicated for immunofluorescence. Representative 60x confocal images are shown. Arrows highlight sites of colocalization.

A

Method of Inhibiting Internalization	Target	2BP-induced c-Met loss
Chlorpromazine	Clathrin Pit Formation	no change
C3 Transferase	RhoA,B,C	no change
Vinblastine	Microtubules	no change
Y27632	ROCK I, II	no change
Latrunculin	Actin	no change
Dansylcadaverine	Clathrin Pit Formation	no change
EIPA	Endosomal pH gradient	no change
Hyperosmotic Sucrose	Clathrin Pit Formation	no change
mβ-cyclodextrin	Lipid Raft	no change
Nystatin	Lipid Raft	no change
CHC siRNA	Clathrin Pit Formation	no change
Dynamin siRNA	Dynamin-Dependent Scission	no change

Supplementary Figure S3: 2BP-induced c-Met loss is not dependent on rapid internalization. A. DU145 cells were treated with 100 μM 2BP for 6 hours in the presence or absence of the indicated inhibitors. Inhibitors were used at maximum tolerable concentrations. Alternatively, cells with confirmed knockdown of clathrin heavy chain (CHC) or dynamin were treated with 2BP. Western blot analysis was performed to determine levels of c-Met. A significant change was considered a greater than 20% reduction in 2BP-induced c-Met loss.

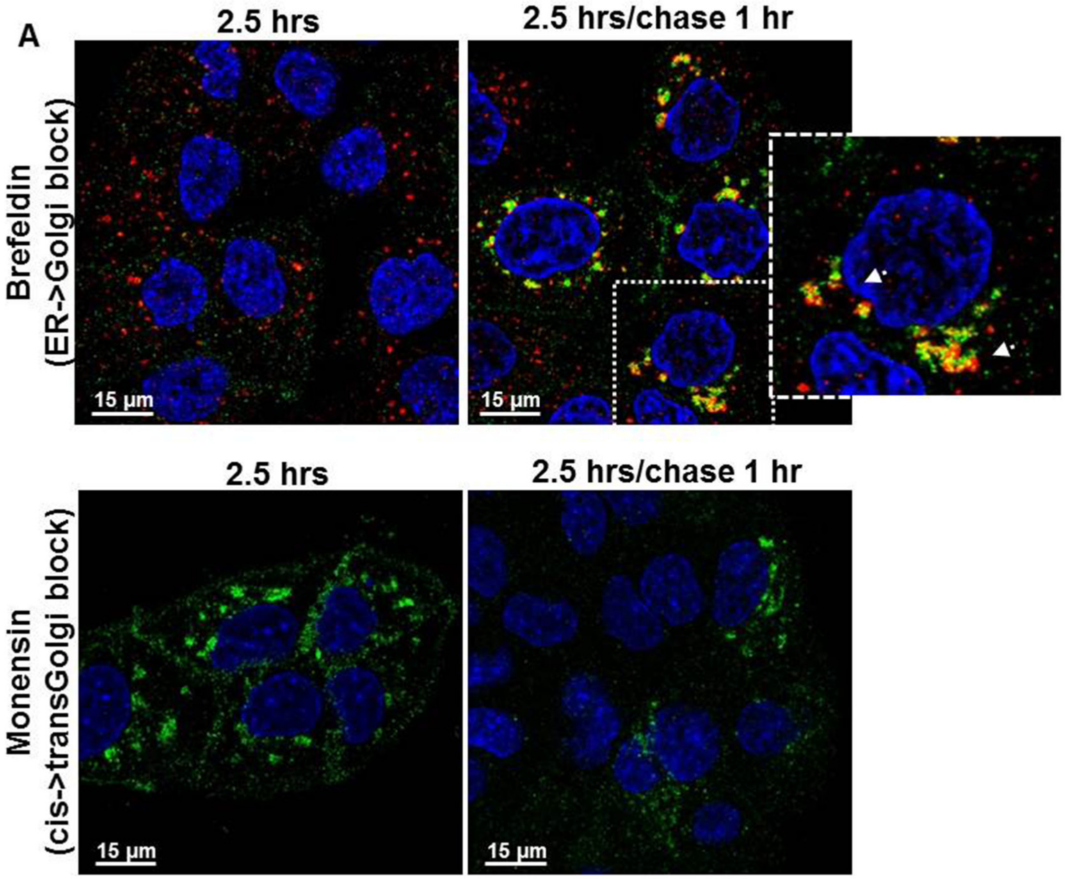

Supplementary Figure S4: Characterization of brefeldin A and monensin treatment on c-Met subcellular distriubution and proteolytic processing. DU145 cells were treated with **A.** 2 µM Brefeldin A (top), a reversible inhibitor of ER eggress, or 2 µM monensin (bottom), a reversible inhibitor of cis- to trans-Golgi trafficking, for 2.5 hours prior to fixing. Alternatively, cells were treated for 2.5 hours then washed for an additional hour chase period. The antibody used for c-Met (green) detection by immunofluorescence was not able to detect precursor c-Met, but following the removal of brefeldin A, the mature form is detected emerging in the cis-Golgi (red) having been processed. Representative 60x confocal images are shown. Arrows highlight sites of colocalization.
